# The effects of a novel personal comfort system on thermal comfort, physiology and perceived indoor environmental quality, and its health implications ‐ Stimulating human thermoregulation without compromising thermal comfort

**DOI:** 10.1111/ina.12951

**Published:** 2021-11-01

**Authors:** Wei Luo, Rick Kramer, Yvonne de Kort, Pascal Rense, Wouter van Marken Lichtenbelt

**Affiliations:** ^1^ Department of Nutrition and Movement Sciences School of Nutrition and Translational Research in Metabolism Maastricht University Maastricht The Netherlands; ^2^ Department of the Built Environment Eindhoven University of Technology Eindhoven The Netherlands; ^3^ Department of Industrial Engineering and Innovation Sciences Eindhoven University of Technology Eindhoven The Netherlands

**Keywords:** drifting temperature, health, indoor air quality, personal comfort system, thermal comfort, thermoregulation

## Abstract

The classical textbook interpretation of thermal comfort is that it occurs when the thermoregulatory effort is minimized. However, stimulating human thermoregulatory systems may benefit health and increase body thermal resilience. To address this gap, we tested a novel personal comfort system (PCS) that targets only the extremities and the head, leaving the rest of the body exposed to a moderately drifting temperature (17–25°C). A randomized, cross‐over study was conducted under controlled laboratory conditions, mimicking an office setting. Eighteen participants completed two scenarios, one with a PCS and another one without a PCS in 17–25°C ambient conditions. The results indicate that the PCS improved thermal comfort in 17–23°C and retained active thermoregulatory control. The torso skin temperature, underarm‐finger temperature gradients, energy expenditure, substrate oxidations and physical activity were not affected by the PCS in most cases. Only slight changes in cardiovascular responses were observed between the two scenarios. Moreover, the PCS boosted pleasure and arousal. At 25°C, the PCS did not improve thermal comfort, but significantly improved air quality perceptions and mitigated eye strain. These findings suggest that human physiological thermoregulation can be stimulated without compromising thermal comfort by using a PCS that only targets the extremities in cold conditions.

**Practical Implications**

The designed PCS achieved an 84% comfortable rate in a drifting temperature scenario over a wide range of ambient air temperatures (17–25°C), which potentiates significant energy savings. Moreover, a positive, healthy thermal stimulus to the body can be sustained. The designed PCS, therefore, implies a great potential for the future to create a healthy, comfortable and energy‐efficient built environment.

## 1 INTRODUCTION

Humans spend 80%–90% of their time indoors.^1^, ^2^ Hence, indoor environmental conditions have a fundamental impact on human health, comfort and productivity. The primary purpose of indoor temperature control is to provide thermal comfort, the so‐called “condition of mind that expresses satisfaction with the thermal environment”.^3^ The general notion is that thermal comfort occurs when body temperatures are kept within a small range to minimize the thermoregulatory effort of the body.^4^, ^5^, ^6^ Hence, in the past decades the paradigm has developed that indoor temperatures in air‐conditioned buildings should be maintained within a stringent temperature range with limited permissible daily and seasonal variations. This paradigm is mainly caused by strict interpretations of the Predicted Mean Vote (PMV) model in the ASHRAE standards^3^ and ISO standards,^7^ which are based on the work of Fanger.^8^

Indoor climate conditioning is associated with 20% of the global energy consumption.^9^, ^10^ Driven by sustainability challenges and climate change, it is worthwhile to start rethinking the current paradigm of strict indoor climate control. In this context, a highly relevant question is whether such a narrow indoor temperature range is beneficial for human health or not. Previous studies^11^, ^12^ show that several metabolic diseases may be related to permanently residing in constant thermal neutral conditions. Furthermore, literature suggests a causal link between people mostly residing in the thermoneutral zone (TNZ) and the increased prevalence of obesity.^13^, ^14^ The TNZ is defined as the range of ambient temperatures without regulatory changes in metabolic heat production or evaporative heat loss.^15^ Kingma et al.^16^ suggest that the thermal comfort zone in a uniform and stable condition is a subset of the TNZ. Therefore, the prevalent stable indoor climate design may keep people in the TNZ minimizing energy metabolism, which may, in combination with other lifestyle factors, tip down the energy balance to the ‘gaining weight’ side.^17^, ^18^ Variations outside the TNZ can increase the metabolism and reverse the ‘tipping’.^19^ Moreover, regularly exercising thermoregulation in mild cold/heat can benefit body glucose metabolism (increase insulin sensitivity)^20^, ^21^ and reduce the risks of cardiovascular diseases.^22^, ^23^ Van Marken Lichtenbelt et al.^19^ document the details of those health implications in their review paper.

Another concern of the current paradigm of stable indoor climate design is possible decreased body thermal resilience, that is, our ability to cope with (extreme) non‐neutral conditions. The current indoor temperature design minimizes the thermoregulatory effort, which means less or no “stimulation” to the thermoregulation system, jeopardizing our thermal resilience. Thermal resilience is even more of interest in the context of global warming with increased likelihood of more extreme weather events.^24^, ^25^ Hence, another argument to rethink the current paradigm is that regularly stimulating thermoregulation in mild cold/heat increases thermal resilience,^26^ mitigating physiological stress in extreme conditions.

The adaptive thermal comfort model by de Dear et al.^27^ may have the potential to bridge health and thermal comfort as it allows for much more temperature variation, both diurnal and seasonal. Based on field study data in naturally ventilated buildings, the adaptive thermal comfort model established a linear relationship between a comfortable temperature range and running mean outdoor temperature of the past few days.^27^ The adaptive thermal comfort model proposes a combination of three underlying mechanisms: (i) behavioral adaptation such as clothing adjustments, (ii) physiological adaptation and (iii) psychological adaptation.^28^ However, behavioral adaptation may substantially reduce thermal stress to the extent where thermoregulatory efforts are limited, and therefore physiological adaption may not occur.

Personal comfort systems (PCSs) in combination with varied ambient thermal conditions, even beyond adaptive thermal comfort limits, have the potential to provide climatic conditions near occupants that are favorable for health while fulfilling the need of thermal comfort. A PCS directly targets human body segments, such as hands, feet and the torso, using person‐controlled local heating/cooling. This close‐to‐body conditioning provides individual thermal comfort while relaxing ambient conditions to a range of 14°C–32°C,^29^, ^30^ and, hence, paves the way for substantial building energy savings.^29^, ^30^ More essentially, a PCS can precisely eliminate the local thermal discomfort of targeted body segments while the thermal stimulus to other untargeted body segments may remain, which may activate body thermoregulation and benefit health as outlined above. However, most of the studies only address the effect of PCSs on thermal comfort and energy savings (see recent reviews by Zhang et.al.^29^ and by Veselý et.al.^30^) and the majority employs torso heating/cooling or conditioning of large body areas.^31^, ^32^, ^33^ Thereby, the thermal stimulus to the body may be reduced substantially. This study focuses on a novel PCS concept that targets only the extremities and the head to maintain the thermal stimulus to elicit the above‐mentioned health benefits. Previous literature indicates that the hands and feet are the most uncomfortable body parts in mild cold and that the head is the most uncomfortable part in warm conditions.^34^, ^35^ Therefore, the novel PCS precisely targets local thermal discomfort of the extremities and the head, to improve overall thermal comfort while maintaining the thermal excitation to the torso in mild cold/warm conditions. This may provide a solution for the future to create a healthy, comfortable and energy‐efficient environment.

This study hypothesizes that human thermoregulation can be stimulated in a moderately drifting thermal environment while providing comfort using a PCS design that targets only the extremities and the head. Therefore, this study investigates the effects of the designed PCS system on (1) thermal comfort, (2) human physiology (including thermoregulatory, cardiovascular and metabolic responses), and (3) perceptions of indoor environmental quality (including air quality, eye symptoms and experienced pleasure and arousal).

## 2 METHOD

### 2.1 Participants

Eighteen healthy participants were recruited (Caucasian, nine males and nine females), 18–40 years old with a BMI of 18–27.5 kg/m^2^ (Table 1). Female participants were taking contraception and the measurements were conducted after their menstrual cycle to minimize hormonal influences. Smokers, individuals with Raynaud's phenomenon, and people who are taking medication that will interfere with outcome parameters were excluded.

**TABLE 1:** Participants’ characteristics

	Mean (±SD)
N	18
Age (year)	22.3 ± 3.4
Height (m)	1.61 ± 0.41
Weight (kg)	67.0 ± 8.3
BMI (kg/m^2^)	23.0 ± 1.8
Fat mass (%)	24.6 ± 7.3
Body surface area (m^2^)	1.78 ± 0.16

### 2.2 Experimental protocol

This study was performed in conformity with the Declaration of Helsinki (Fortaleza, Brazil, 2013) at Maastricht University from November 2019 to March 2020 and from September 2020 to October 2020 (winter and autumn seasons in the Netherlands). The study was approved by the Medical‐ethical committee of Maastricht University and was registered at the Netherlands Trial Registry (number: NL7757).

#### 2.2.1 Personal comfort system

**FIGURE 1:**
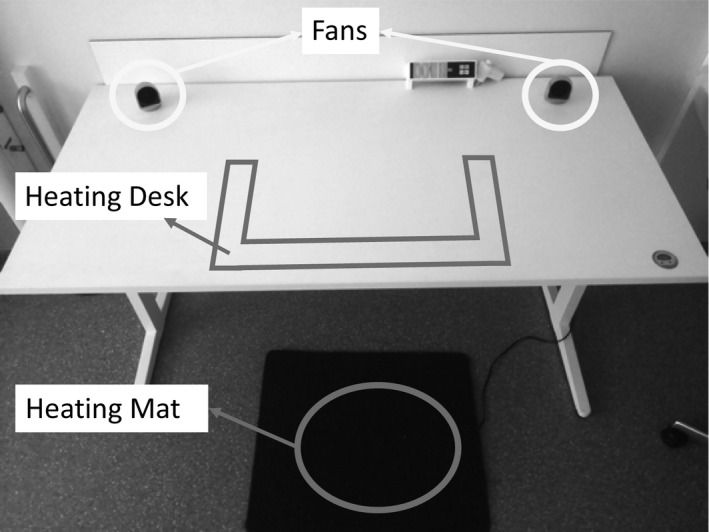
Designed personal comfort system including two ventilation fans (yellow ovals), a heating desk and a heating mat. The red areas indicate the heating areas

The PCS was designed to warm up the extremities and cool down the head. It includes a heating desk, a heating mat on the floor and two fans for ventilation (Figure 1). Each device has four settings: off, low, medium and high (for surface temperatures and air speed rates see Table 2). The participants were able to control the devices at their desires in the PCS scenario. The settings of the PCS were monitored and recorded. In addition, the airflow direction of the ventilation fans was adjusted before the experiment, to direct the air flow on the participant's head area. It remained in that direction during the experiment.

**TABLE 2:** Setting specifications of the designed personal comfort system

	Off	Low	Medium	High
Heating desk (surface temperature)	/	31°C	34°C	37°C
Heating feet mat (surface temperature)	/	36°C	41°C	46°C
Ventilation fans (air speed rate at 80 cm away from the ventilator)	/	0.3 m/s	0.6 m/s	1.2 m/s

#### 2.2.2 Study design

**FIGURE 2:**
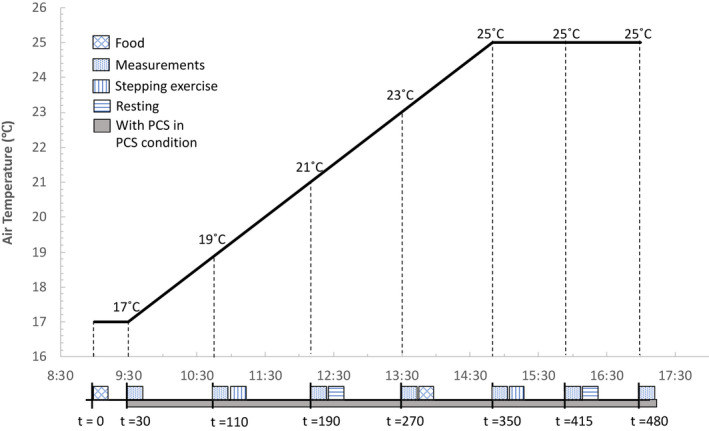
Protocol of the experiment

This study has a cross‐over, randomized design, consisting of two scenarios in drifting ambient temperature conditions (Figure 2) on two separate days ‐ with PCS (PCS scenario) and without PCS (NOPCS scenario). Participants started either with PCS scenario or NOPCS scenario based on a permuted blocked randomization procedure. To avoid possible short‐term thermal acclimation induced by the experiment's exposure,^36^ the two test days were at least one day apart. To minimize the influence of long‐term seasonal thermal acclimatization,^37^ the two test days were scheduled within 14 days. Throughout the study, participants were not informed about the actual ambient temperature.

An office setting was emulated in the climate chambers of the Metabolic Research Unit of Maastricht University, where participants stayed for eight hours (from 9:00 h to 17:00 h) and performed typical office work (activity level ≈ 1.2 METs). In the chamber, participants wore standardized clothing, including underwear, a long‐sleeved shirt, sweatpants, socks and shoes and sat on an office chair (total of 0.80 clo). Whereas most of the PCS studies were conducted in static environments, this PCS was tested in a dynamic environment to provide new insights regarding PCS’s performances in dynamic environments. Moreover, a dynamic environment may reduce building energy consumption.^38^ Our research group has previously demonstrated that drifting temperatures (17–25°C) stimulate humans’ thermoregulations^39^ and will not lead to unacceptable conditions.^40^ Therefore, a dynamic profile was applied to the climate chamber's indoor temperature starting at 17°C for 30 min, followed by a 1.5°C/h ramp for 320 min, and remaining stable at 25°C for 130 min (Figure 2). The designed minimum temperature of 17°C was intended to avoid shivering.^40^ The maximum temperature of 25°C fits the thermal comfort zone (PMV is around 0.5) according to ASHRAE standard^3^ and ISO standard^7^. The imposed ramp of 1.5°C/h is within the ISO 7730 comfort ramp limit.^7^ According to earlier studies,^40^, ^41^ thermal sensation under the imposed slow ramp should not be affected by the previous thermal experience. An up ramp was chosen as it follows the rising trend of outdoor temperatures during office hours in winter (9:00–17:00), and, hence, is in line with the drifting temperature pattern that would occur in practice to improve energy efficiency in the office buildings. The first 30 min at 17°C intended to let participants habituate to the cold environment. The maximum temperature of 25°C lasted for two hours to achieve a stable thermal state as in step‐change transient temperatures the thermal sensation stabilizes after 30 min.^42^

#### 2.2.3 Standardization before test days

The participants attended a standardization session one day before every test day to acquaint themselves with the climate chamber, the standardization procedure and the test procedure. To stabilize the participants’ physiological state during the test day, the lifestyles were standardized 24 h before the test day. Participants were asked to consume the same amount and kind of food as much as possible in the two standardization sessions. They were also asked to refrain from taking food and drinks after 22:00 h with the exception of water, and to stick to normal bedtimes and sleep duration in the night before the test day. Furthermore, participants were instructed to avoid any moderate to vigorous exercise, caffeine or alcohol a day before the measurements. To discourage strenuous physical activity, the participants wore an activity monitor (MOX, Maastricht Instruments, the Netherlands) to check their physical activity a day before the measurements. Additionally, before the PCS scenario, participants were familiarized with the PCS for at least an hour as this study focuses on the long‐term usage of PCS.

#### 2.2.4 Procedure during test days

During the test days, participants arrived at 8:00 h in the morning after an overnight fast and travelled to the laboratory with light physical activity. Upon arrival, participants got dressed in standardized clothing (0.80 clo) and resided in a respiration chamber at 23°C (thermal neutral environment). Wireless skin temperature sensors, a hand skin blood flow sensor, a blood pressure monitor cuff and a heart rate chest belt were placed. The physical activity monitor remained with participants during the test day. Later, participants rested on a bed for 30 min to measure the basal metabolic rate (BMR), during which blood pressure and hand skin blood flow were measured as well.

Afterward, participants moved to a climate chamber maintained at 17°C for 30 min followed by a temperature ramp of 1.5°C/h. In the meantime, participants were allowed to use the PCS at liberty in the PCS scenario. At every 2°C temperature rise, a blood pressure measurement, a hand skin blood flow measurement and a questionnaire assessing subjective perceptions were taken (*t* = 30 min, 110 min, 190 min, 270 min and 350 min, Figure 2). Once the temperature reached 25℃, these measurements were performed every 65 min (*t* = 415 min and 480 min, Figure 2). Skin temperature, energy expenditure, physical activity and heart rate were continuously measured. Two times during the test day, participants performed a stepping exercise for 5 min to emulate normal office walking activity. Participants rested twice in a supine position for 30 min to measure the resting metabolic rate (RMR, Figure 2). Breakfast and lunch were standardized and provided by the researchers on both test days. Water was consumed *at libitum*.

### 2.3 Measurements

#### 2.3.1 Indoor climate measurements

Air temperature was measured during the test days at 0.1 m, 0.6 m and 1.1 m from the ground near the participants using iButton dataloggers (DS1922L, Maxim Integrated, USA). Before the test days, mean radiant temperature, air speed and relative air humidity were also measured using an Almemo 2890–9 device (Ahlborn Mess, Germany). These measurements were done during the same temperature drifts as during the actual measurements with the volunteers. The results indicated that the mean radiant temperature of the test chamber closely followed the air temperature. The air speed was constantly close to 0.2 m/s, and relative humidity remained between 40% and 55% RH.

#### 2.3.2 Participants’ characteristics

The body weight, height and body fat were measured in the morning under a fasted state. Participants’ fat content was determined by an air displacement plethysmograph (Bodpod, Cosmed, Italy). The body surface area was calculated according to the Du Bois formula.^43^

#### 2.3.3 Thermal perception

Based on the ISO standard 10551,^44^ whole‐body thermal sensation, thermal comfort and thermal preference were assessed by visual analogue scales (Figure S1). In addition, thermal acceptance was evaluated using two options (1: ‘Acceptable’ or 0: ‘Unacceptable’). The same thermal sensation scale was used to measure local thermal sensations, including head, neck, front torso, back torso, upper arm, lower arm, hand, thigh, calf and feet. The local thermal sensation of the torso is the average of the back and front torso votes. The local thermal sensation of the extremities is the average of the hands and feet.

#### 2.3.4 Physiological parameters

Sixteen wireless temperature sensors (iButtons DS‐1922 L, Maxim Integrated, USA) were used to measure skin temperature at a sample rate of 1 min. Fourteen body sites were included according to the ISO 9886 standard to calculate the mean skin temperature.^45^ Two additional sensors were placed on the lower arm and middle finger to obtain the underarm‐finger temperature gradient, which is an index for vasomotion.^46^ For the torso skin temperature, the skin temperatures of the lower back, the scapula, the abdomen and the upper chest were averaged. For the distal temperature, the skin temperatures of the hands and feet were averaged.

Basal metabolic rate was measured using indirect calorimetry with a ventilated hood system (Omnical, Maastricht Instruments, the Netherlands). Participants’ energy expenditure was obtained at a sample rate of 5 min during the 8 h exposure by measuring oxygen consumption and carbon dioxide production inside the chamber (Omnical, Maastricht Instruments, the Netherlands). The energy expenditure was calculated according to Weir's equation^47^ and carbohydrate and lipid oxidation were calculated by Péronnet's and Massicotte's formulas.^48^ Physical activity index (PAI) was defined as energy expenditure to BMR ratio. For assessing physical activity at a sample rate of 1 min, a three‐axial accelerometer was placed at 0.1 m above the right patella (MOX, Maastricht Instruments, the Netherlands).

A chest belt was used for measuring heart rate at a sample rate of 1 min (H10, Polar, USA). A cuff was placed on the upper arm to assess the blood pressure (Omron Healthcare B.V., Hoofddorp, the Netherlands). To decrease measurement noise, the blood pressure measurements were repeated three times. The average value of the three measurements was used for further analysis. A Laser Doppler Flowmetry (LDF) was used for measuring skin perfusion at the dorsal side of the non‐dominant hand (PF5000, Perimed AB, Sweden). Since the LDF measures the relative blood flow instead of an absolute blood flow, a normalized blood flow value was determined by dividing the measured value of blood flow during the day with the measured value of blood flow during the BMR measurement.

#### 2.3.5 Perceived air quality, affective states and eye‐related symptoms

For the perceived air quality and air freshness, two continuous scales were used (Figure S2A, B). Moreover, pleasure and arousal components of affect were assessed with the self‐assessment manikin (SAM) scale^49^ (Figure S2C, D). An eye‐related symptoms list was adopted from Viola et al.^50^ using discrete scales (Figure S2E).

### 2.4 Statistical tests

A Mixed Linear Model (MLM) was employed to compare the differences between the PCS and NOPCS scenarios, treating the participants as a random factor. The scenario, measurement timepoints and their interaction were included as fixed factors. To account for the air temperature difference between the two scenarios, the difference between the temperature protocol and actual air temperature was added as well.

To improve the accuracy and increase the power of the MLM, relevant control variables and their interaction with measurement timepoints were considered as covariates: Body surface area to mass, fat‐free mass to body surface ratio, gender and age. For the resting condition, fat and muscle mass play a major role in body insulation, where muscle mass can explain up to 90% of the insulation.^51^ Thus, the mass to body surface area ratio represents the body insulation, whose reciprocal (body surface area to mass) indicates the heat transfer coefficient (heat loss ability). On the other hand, because the fat‐free mass strongly relates to the body's heat production in resting condition,^52^, ^53^ fat‐free mass to body surface ratio indicates the individual heat production ability. Moreover, the thermoregulation ability is also affected by gender and age.^54^, ^55^ In addition, baseline measurements of each test day may indicate daily variations. Therefore, the baseline was included as a covariate as well.

A ‘top‐down’ modelling strategy was used,^56^, ^57^ starting with the maximum model followed by a stepwise backward elimination procedure. The likelihood ratio test was used to compare different models.^57^ Only significant covariates (body surface area to mass, fat‐free mass to body surface ratio, gender, age and baseline) were kept in the model. Once the final model was obtained, the effect of each variable was tested using conditional F‐tests with degrees of freedom correction according to Kenward‐Roger's method. The Kenward‐Roger's method may be more suitable for small sample sizes^58^ and more conservative as compared to the likelihood ratio test.^58^, ^59^

For all continuously measured variables (heart rate, energy expenditure, physical activity and skin temperatures), averages of the data of 10 min before the submission of the questionnaires were used as representative values. During those 10 min, participants were in a more controlled state since participants were sitting on the chair, relaxing, measuring blood pressure and filling in the questionnaire. For the hand skin blood flow, the average of the data during the 5 min keeping still was used. The baseline values were taken from the habituation period (17°C, *t* = 20 min to *t* = 30 min), except for the blood pressure, for which the value during the BMR measurements was used. Assumptions of the MLM were checked: In case the assumption of normality was violated, the non‐parametric paired Wilcoxon signed rank test was used to compare the differences between the two scenarios at each timepoint. The differences in variances were tested using Levene's test.

The open‐source software R Studio 1.3.1073 was used to analyze the data (LmerTest package,^58^ Emmeans package^60^ and Car package^61^). Significance levels <0.05 were treated as significant (* = *p *< 0.05, ** = *p *< 0.01, *** = *p *< 0.001). In all the box plots, the box is defined by the first quartile and the third quartile. The horizontal line in the middle of the box, the dot in the box, and the cross indicate the median, the mean and the outlier, respectively. All the data are reported as mean ± standard deviation, unless stated otherwise.

## 3 RESULTS

### 3.1 Ambient air temperature

The air temperature profiles of both scenarios followed the protocol (Figure S3). A small, but statistically significant difference was observed between the two scenarios: the air temperature was, on average, slightly higher in the PCS scenario. However, the average difference (0.15 ± 0.14°C) is regarded as acceptable.

### 3.2 Thermal perception

**FIGURE 3:**
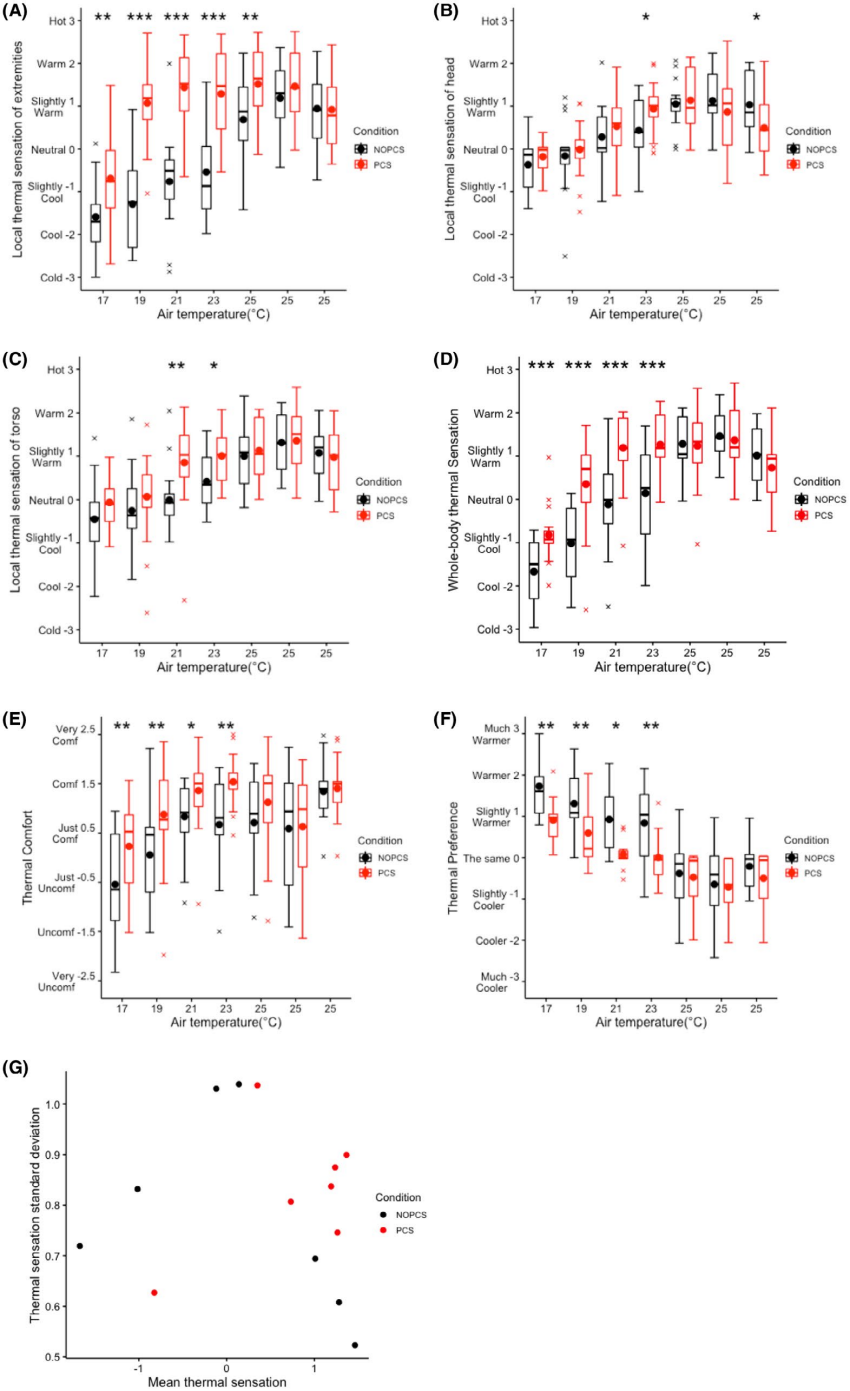
Local and whole‐body thermal perceptions over time: (A) local thermal sensation of extremities, (B) local thermal sensation of head (C) local thermal sensation of torso, (D) whole‐body thermal sensation, (E) thermal comfort, (F) thermal preference, (G) Thermal sensation standard deviation against mean thermal sensation (The thermal sensation's standard deviation near neutral (0) is much higher than the other data points)

Without a PCS, local thermal sensations, whole‐body thermal sensation, thermal comfort and thermal preference were in line with the temperature profile (Figure 3). There were clear individual differences in thermal perception. The standard deviation of thermal sensation was more than 0.5 unit on the 7‐point thermal sensation scale (Figure 3G). Moreover, the individual differences in thermal sensation were more pronounced near the neutral condition while in more extreme conditions, participants’ votes were more consistent (Figure 3G).

Local thermal sensation of the extremities was significantly warmer with the use of a PCS, particularly in the colder part of the temperature ramp (*p *< 0.05, Figure 3A). On the other hand, local thermal sensation of the head was significantly warmer at 23°C, but was significantly lower at the end of 25°C in PCS scenario (*p *< 0.05, Figure 3B). In addition, the thermal sensation of the torso (non‐targeted body segment) was significantly warmer at 21°C and 23°C with a PCS (*p *< 0.05, Figure 3C), while at 17°C, 19°C and 25°C no significant differences were present between the two scenarios (Figure 3C).

As compared to the NOPCS scenario, a PCS resulted in significantly warmer whole‐body sensations, improved whole‐body thermal comfort and lowered heating preference under the cold to neutral environments (17–23°C, *p *< 0.05, Figure 3). In the PCS scenario, 98% of the total votes fell in “acceptable” while in the NOPCS scenario this was 91% of the votes (Table 3). Most importantly, by applying a PCS, the overall comfort rate (equal or higher than “just comfortable”) increased from 71% to 84%, where the increases in the mild cold were most prominent (23% increase at 17°C and 33% increase at 19°C, Table 3). Although the thermal comfort rate was 56% at 17°C with the PCS, the thermal acceptability rate was 89%. In addition, the variances of the thermal preferences in the PCS scenario were significantly lower than in NOPCS scenario at 21°C (*p *< 0.05, Table 3) and showed a trend toward significance at 23°C (*p *= 0.07, Table 3). No significant benefits of the PCS in thermal perception under a slightly warm environment was found, despite the convective head cooling functionality.

**TABLE 3:** Paired comparisons between two scenarios

	17°C (*t* = 30 min)	19°C (*t* = 110 min)	21°C (*t* = 190 min)	23°C (*t* = 270 min)	25°C (*t* = 350 min)	25°C (*t* = 415 min)	25°C (*t* = 480 min)	Average
Thermal acceptability rate	67% vs. 89%	94% vs. 94%	100% vs. 100%	100% vs. 100%	89% vs. 100%	89% vs. 100%	100% vs. 100%	91% vs. 98%
Thermal comfort rate	33% vs. 56%	56% vs. 89%	89% vs. 94%	78% vs. 100%	78% vs. 83%	67% vs. 72%	94% vs. 94%	71% vs. 84%
Thermal preference standard deviation	0.69 vs. 0.57	0.69 vs. 0.86	0.77 vs. 0.39 ^*^	0.95 vs. 0.63 ^†^	0.93 vs. 0.70	0.94 vs. 0.74	0.55 vs. 0.72	/

Note

Thermal acceptability rate is the percentage of ‘acceptable’ votes. Thermal comfortable rate is the percentage of votes equal or higher than ‘just comfortable’. The statistics of thermal preference standard deviation are based on Levene's test comparing two scenarios. The data are shown as NOPCS scenario vs. PCS scenario.

†*p *= 0.07

### 3.3 Physiology

#### 3.3.1 Skin temperature

**FIGURE 4:**
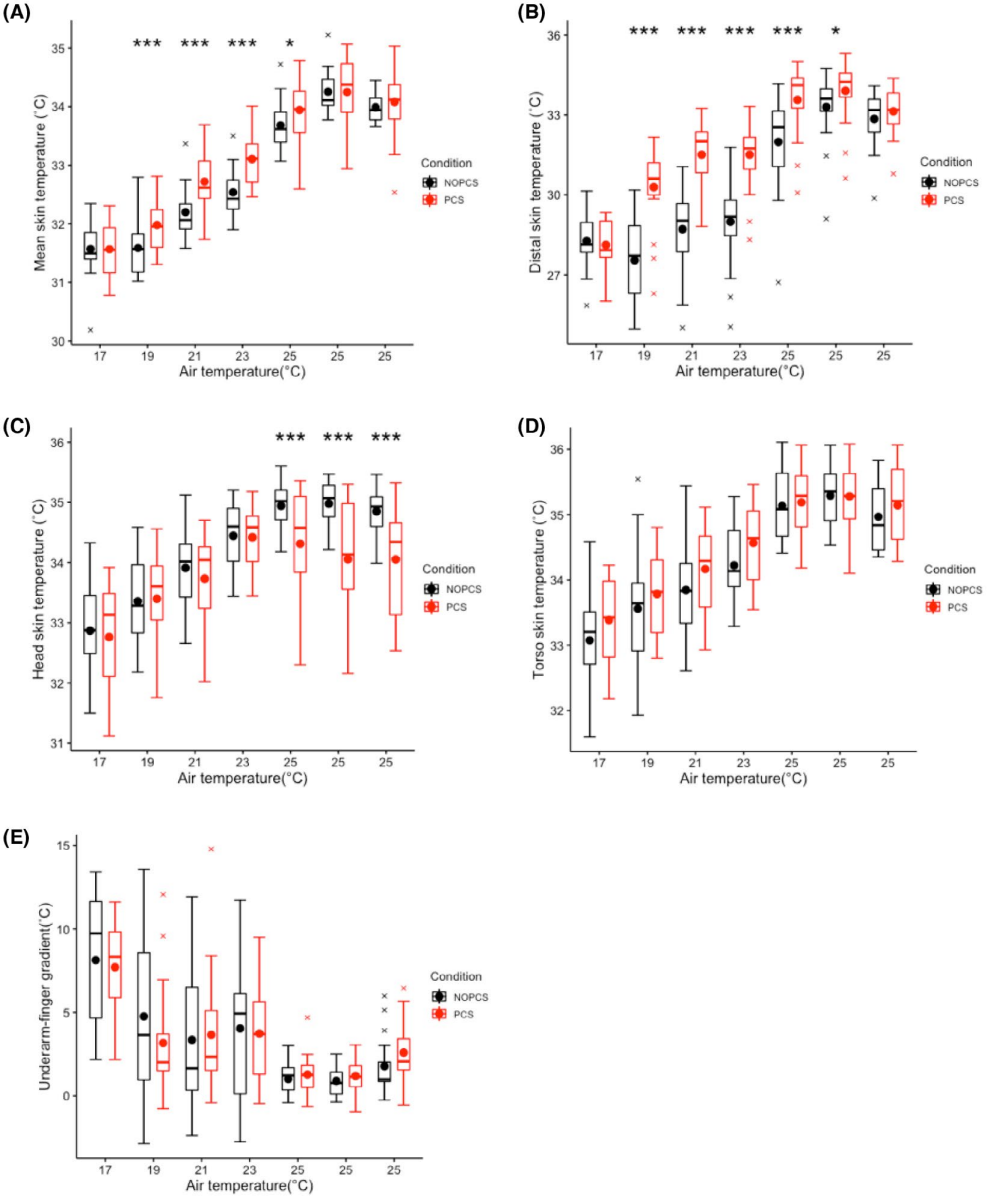
Body skin temperatures over time: (A) mean skin temperature, (B) distal skin temperature, (C) head skin temperature, (D) torso skin temperature, (E) underarm‐finger skin temperature gradient

The mean skin temperature, distal skin temperature, head skin temperature, torso skin temperature and underarm‐finger gradient generally followed the temperature profile in both scenarios (Figure 4). The underarm‐finger temperature gradient is an index for vasomotion. From 17 to 23°C of the NOPCS scenario, the ranges (max‐min) of underarm‐finger gradient were 11, 16, 14 and 14°C respectively (Figure 4E), which indicates large physiological individual differences in vasomotion.

As compared to the NOPCS scenario, the mean skin temperature was significantly higher in the PCS scenario at 19, 21, 23°C and at the beginning of 25°C (*p *< 0.05, Figure 4A). On average, mean skin temperature increased 0.26 ± 0.5°C by using a PCS. The distal skin temperature in the PCS scenario was significantly higher at 19, 21, 23 and the first 25°C‐time point (Figure 4B) while the head skin was significantly lower at all time points at 25°C (Figure 4C). No significant differences in torso skin temperature and underarm‐finger gradient between the two scenarios were found (Figure 4D, E).

#### 3.3.2 Cardiovascular responses

**FIGURE 5:**
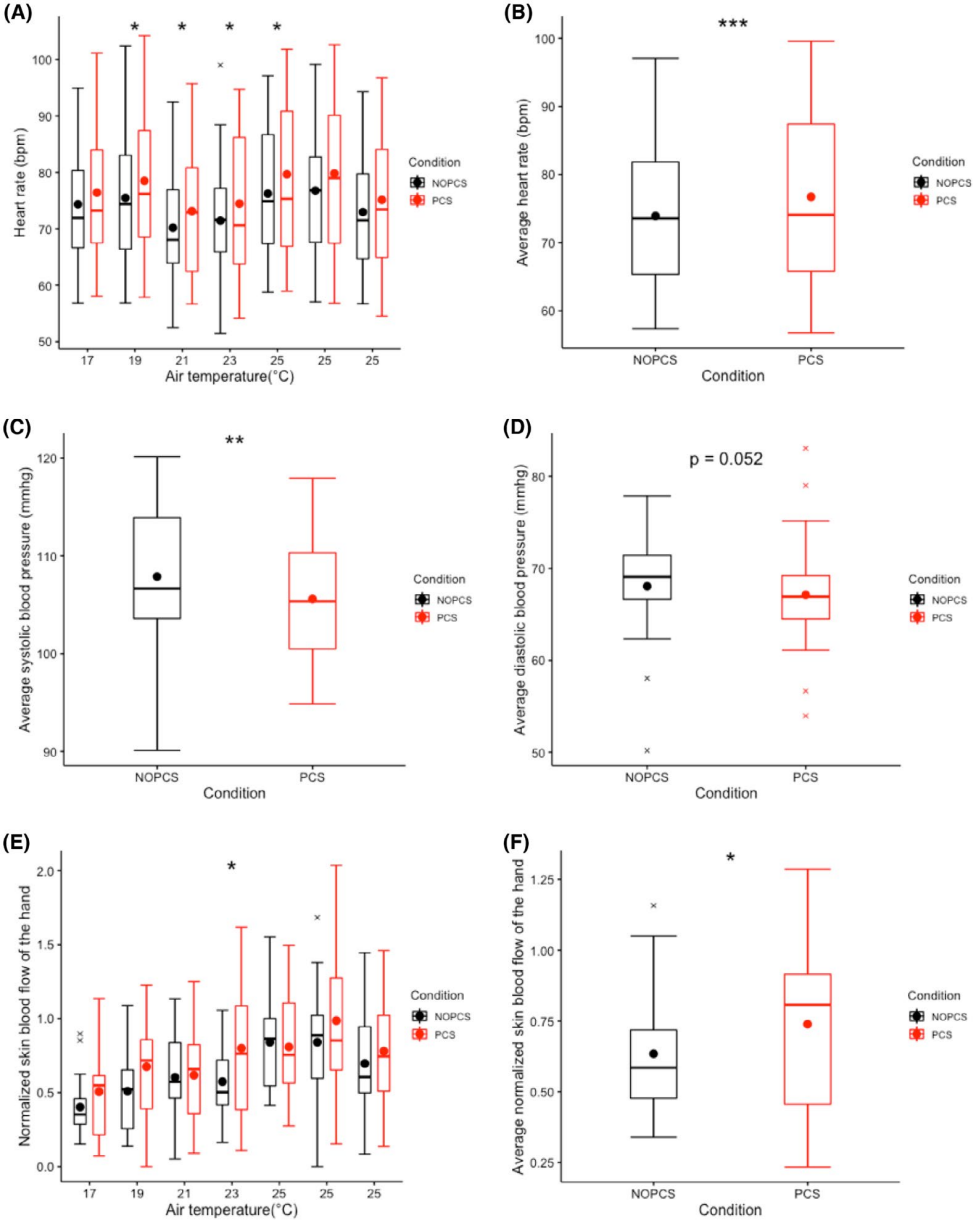
Cardiovascular responses: (A) heart rate over time, (B) average heart rate of all measurements, (C) average systolic blood pressure of all measurements (D) average diastolic blood pressure of all measurements (E) normalized hand skin blood flow over time (F) Average normalized hand skin blood flow of all measurements

Heart rate over time shows no clear relation with the temperature ramp (Figure 5A). However, the hand skin blood flow increased with increasing ambient temperature (Figure 5E). The PCS significantly increased the average heart rate by 2.2 bpm (*p *< 0.001, Figure 5B), decreased systolic blood pressure by 1.5 mmHg (*p *< 0.01, Figure 5C), and diastolic blood pressure by 0.9 mmHg (*p* = 0.052, Figure 5D). The hand skin blood flow was significantly higher at 23°C in the PCS scenario (*p *< 0.05, Figure 5E) while no significant differences were found at other temperatures (Figure 5E) between the two scenarios. Moreover, the PCS significantly elevated the average hand skin blood flow by 8% (*p *< 0.05, Figure 5F).

#### 3.3.3 Energy expenditure, substrate oxidation and physical activity

**FIGURE 6:**
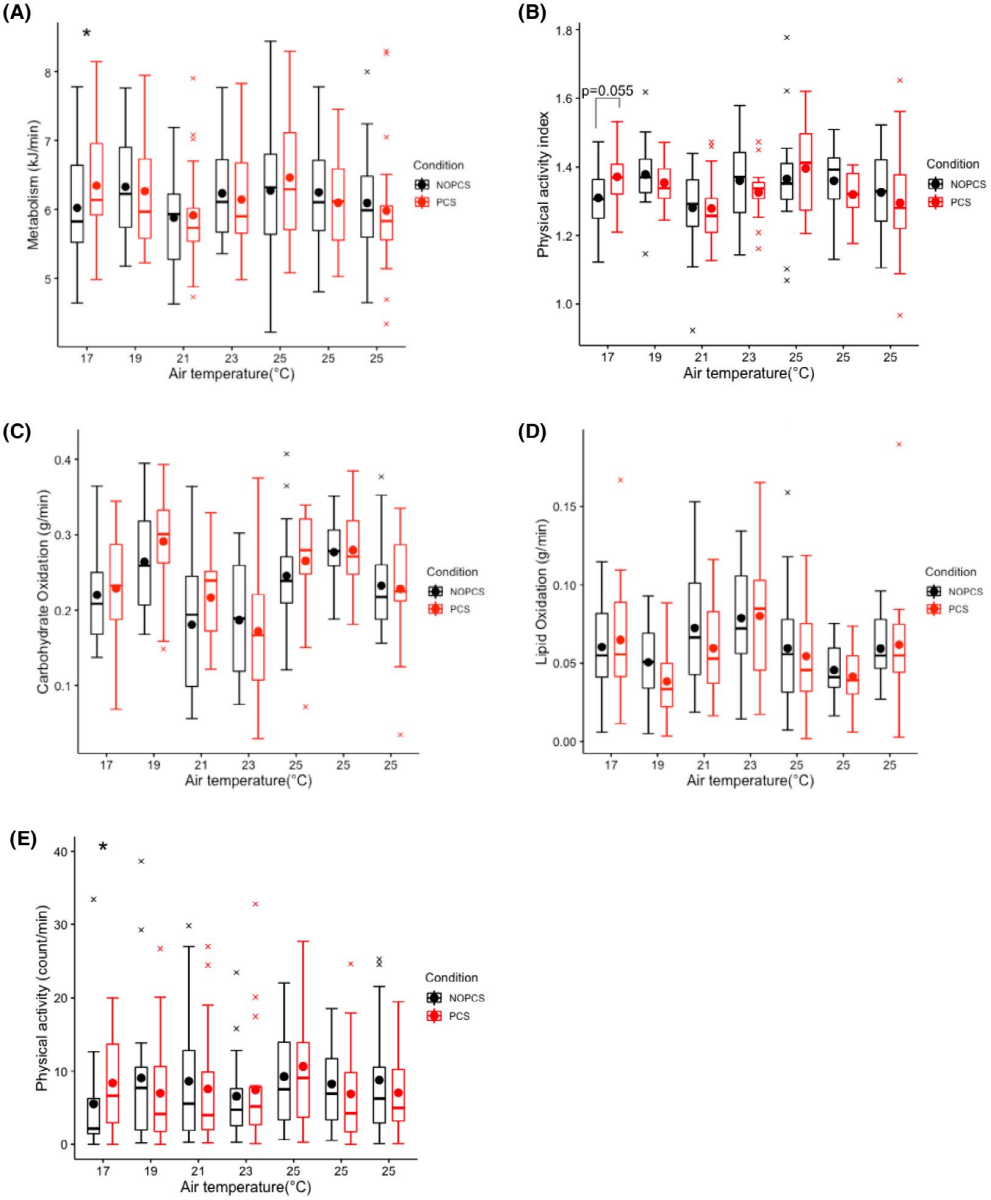
Energy expenditure, substrate oxidation and physical activity over time: (A) energy expenditure, (B) physical activity index, (C) carbohydrate oxidation, (D) lipid oxidation, (E) physical activity

The energy expenditure was significantly higher at 17°C in the PCS scenario than in the NOPCS scenario (*p *< 0.05, Figure 6A). The average difference was 0.32 kJ/min, which is equivalent to 6% of BMR. PAI tended to be larger in PCS scenario at 17°C (*p *= 0.055, Figure 6B). In line, physical activity measured by accelerometry was also found to be significantly higher at 17°C (*p *< 0.05, Figure 6E). Carbohydrate and lipid oxidations were similar between the two scenarios among all temperatures (Figure 6C, D).

### 3.4 Air quality perception, eye‐related symptoms and affective states

#### 3.4.1 Air perception

**FIGURE 7:**
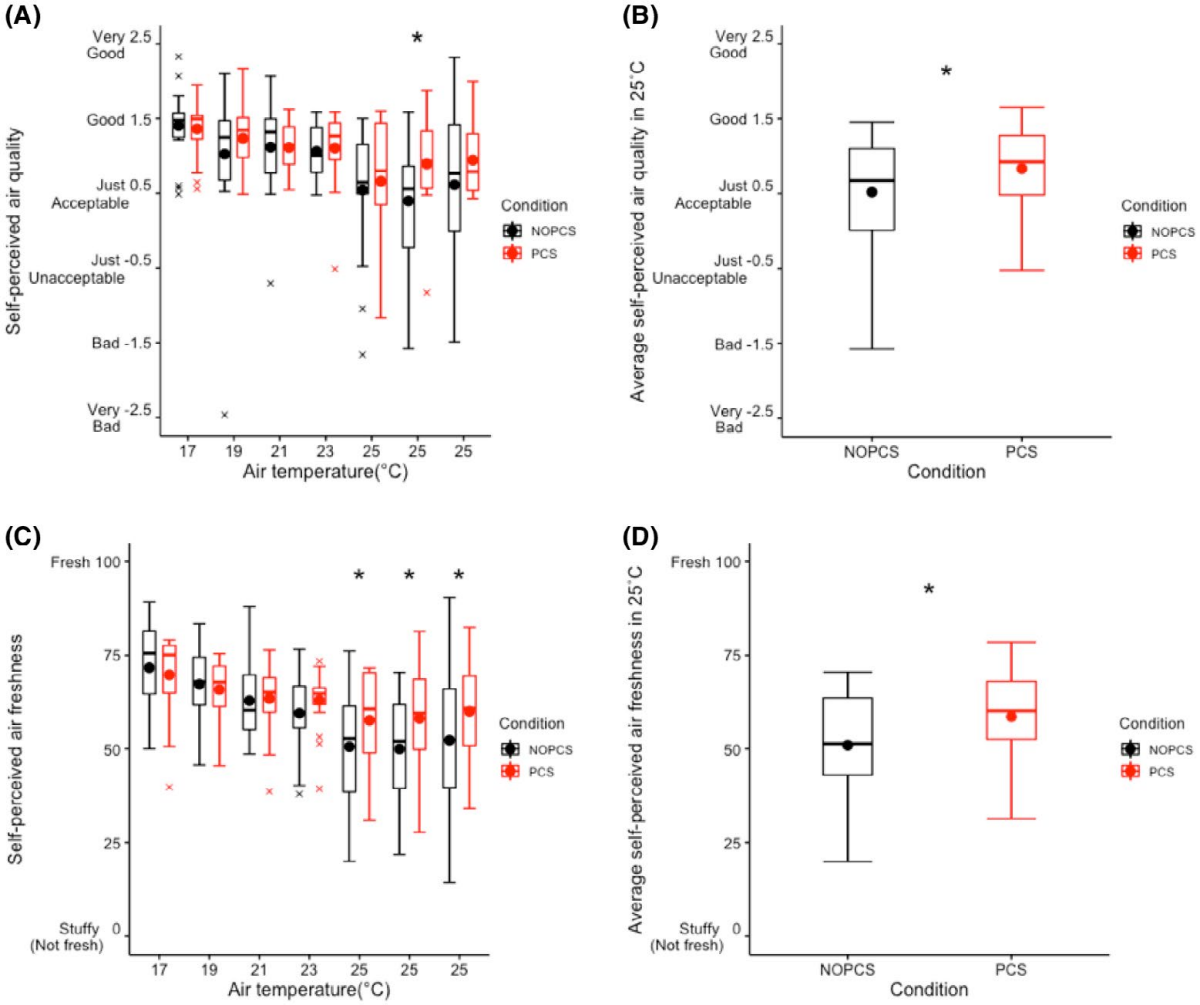
Air quality perception: (A) self‐perceived air quality over time, (B) average self‐perceived air quality of three measurements (*t* = 350 min, 415 min and 480 min) at 25°C, (C) self‐perceived air freshness over time, (D) average self‐perceived air freshness of three measurements (*t* = 350 min, 415 min and 480 min) at 25°C

Overall, both perceived air quality and air freshness were negatively related to the air temperature (*p *< 0.05, Figure 7A, C). No difference was found between the two scenarios in cold and neutral environments (17–23°C, Figure 7A, C). Interestingly, the PCS significantly improved average perception of air quality (0.3 ± 0.6 unite increase, *p *< 0.05 Figure 7B) and air freshness (7.6 ± 15.4 unite increase, *p *< 0.05, Figure 7D) at 25°C.

#### 3.4.2 Eye‐related symptoms

**FIGURE 8:**
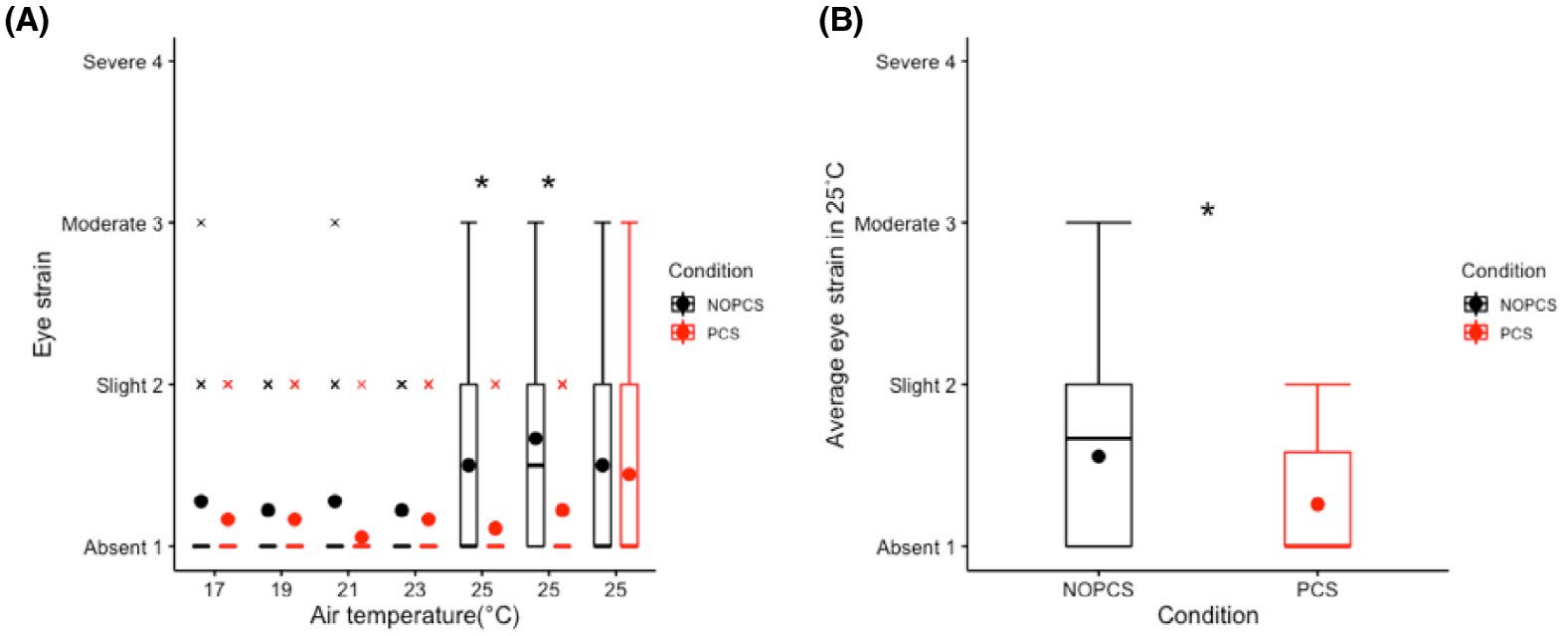
Eye strain symptom: (A) eye strain over time (B) average eye strain of three measurements (*t* = 350 min, 415 min and 480 min) at 25°C

Most votes of the eye discomfort, eye fatigue and eye strain symptoms were no more than “Slight” (Figure 8 and Figure S4). Eye discomfort and eye fatigue appeared to be unaffected by PCS throughout time (Figure S4A, B). However, the PCS significantly reduced average eyestrain at 25°C (*p *< 0.05, Figure 8B), while at 17–23°C, no significant effect of the PCS on eye strain was found (Figure 8A).

#### 3.4.3 Affective states

**FIGURE 9:**
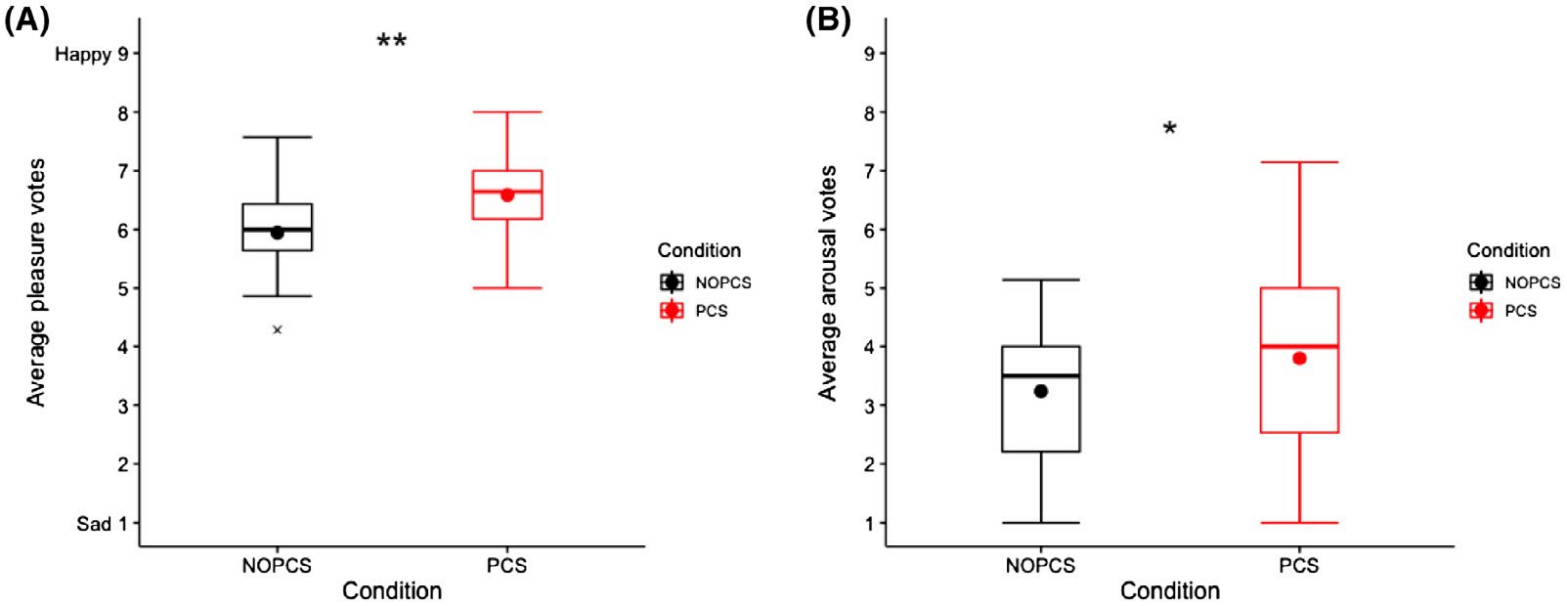
Average affective state vote of all measurements: (A) pleasure (B) arousal

Pleasure and arousal levels remained relatively stable during the day (Figure S5A, B). However, the average votes of all measurements in the PCS scenario generally indicated more pleasure, and more arousal than in the NOPCS scenario (*p *< 0.05, Figure 9A, B).

## 4 DISCUSSION

This study investigated the effects of a novel PCS affecting distal parts of the body, on thermal comfort, physiology and perceived indoor environmental quality in a moderately drifting ambient temperature. We demonstrated that the designed PCS significantly improved thermal comfort in an ambient temperature range of 17–23°C. The distal skin temperatures and head skin temperature were significantly affected by using the PCS. However, the torso skin temperature and underarm‐finger temperature gradient were similar between the PCS and NOPCS scenarios. PCS usage resulted in higher energy expenditure and physical activity at 17°C. At the other temperatures, the PCS did not significantly influence the energy expenditure, substrate oxidation and physical activity. Regarding the cardiovascular responses, a significant but small increase in hand skin blood flow, higher heart rate and lower blood pressure were found in the PCS scenario. In addition, the PCS boosted pleasure and arousal. Although at 25°C the PCS did not improve thermal comfort, it significantly improved perceptions of air quality and air freshness, and mitigated eye strain.

### 4.1 Individual differences, thermal perception and control behavior

**FIGURE 10:**
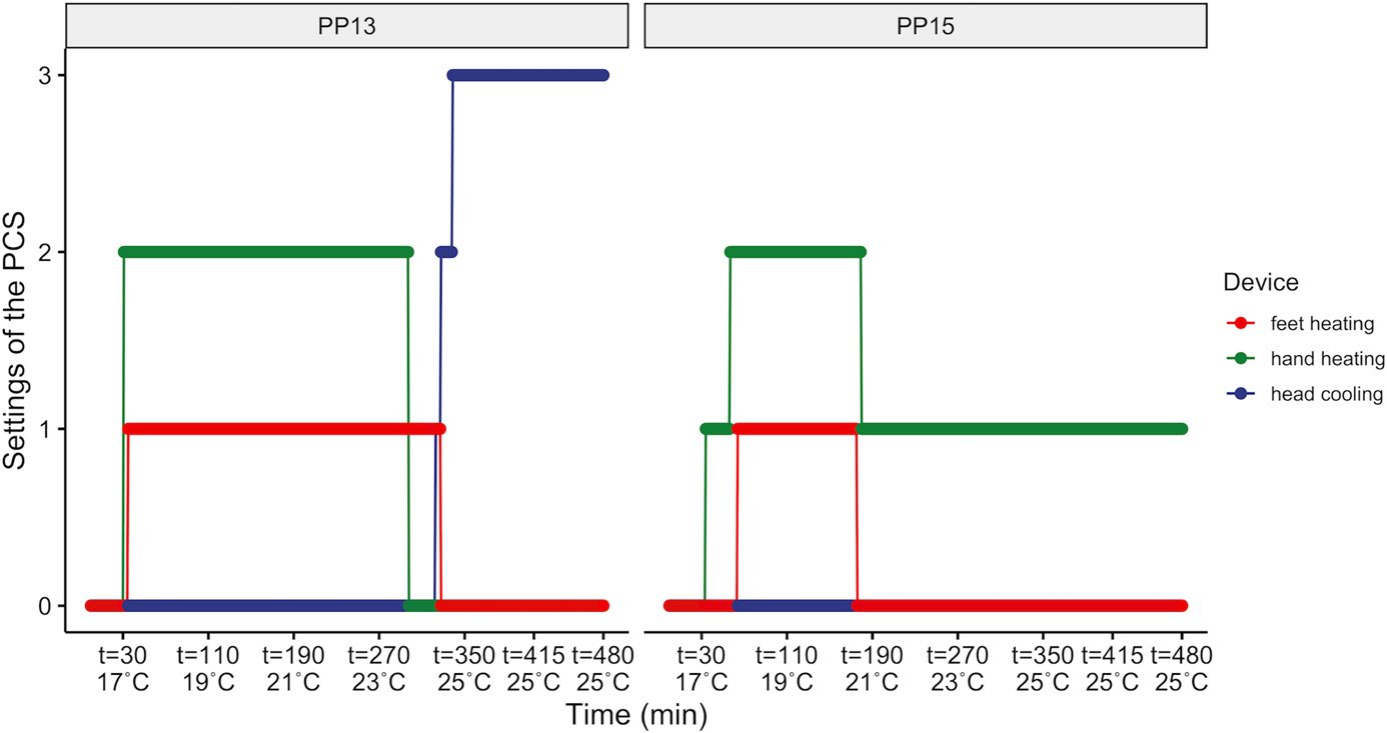
Two participants’ PCS‐using behavior

This study indicates that individual differences in thermal perceptions are prominent, also after controlling for BMI and age. The individual differences in thermal sensation were more prominent near thermal neutrality (Figure 3G). This finding is consistent with Luo's study,^62^ indicating that the participants’ thermally perceptive responses are more diverse near the neutral condition. Furthermore, our results showed that, by using the PCS, participants preferred less change in their thermal environment and the variance of the thermal preference votes was significantly smaller near‐neutral conditions (21 and 23°C, Table 3). Counter‐intuitively, this implies that the personal control mode can contribute more to individual differences near the neutral condition, as the participants have a larger variance in thermal demands. Figure 10 further illustrates how the individual differences in near‐neutral conditions were compensated by the PCS by control behavior adjustments between two selected participants. During 17–21°C, those two participants had similar heating settings, while near the neutral condition, 21–25°C, the two participants present quite distinct control behavior.

In this study, we attempt to mitigate thermal discomfort by targeting the most uncomfortable body segments. As expected, the local skin temperature of the targeted body segments (hand, feet and head) were significantly affected by the use of the PCS (Figure 4B, C). Accordingly, the local thermal perceptions were significantly affected (Figure 3A, B). On a whole‐body level, the designed PCS significantly improved the thermal comfort in the range of 17–23°C (Figure 3C) and achieved 80% thermally comfortable rates in general. This outcome is contrary to that of Veselý et al.,^63^ who found that the thermal discomfort was barely mitigated by merely warming up hands or feet at 18°C. The divergence could be explained by the complaint‐driven feature in previous studies,^34^, ^64^, ^65^ indicating that one or two of the most uncomfortable body segments dominate the whole‐body thermal comfort. Heating hands or feet separately in Veselý’s study^63^ could not solve the two main local discomfort sources, that is, hands and feet in cold conditions,^34^, ^35^ whereas our system targeted both sites simultaneously, thereby mitigating all main local discomfort and thus improving whole‐body thermal comfort. Our result is also supported by the work of Zhang,^66^ finding a significant improvement of thermal comfort by warming up wrists and feet together in cold conditions. Surprisingly, cooling the head did not significantly improve thermal comfort in 25°C, which is against the findings in the study by Nakamura^67^ and the study by Pallubinsky.^68^ The difference in environmental temperatures between our study and those two studies can possibly explain this inconsistency. The warm condition in our study was 25°C while it was 32–34°C in the other studies.^67^, ^68^ In the current study, the participants felt comfortable and preferred no change in 25°C (Figure 3F), which suggests that there was little to improve. The fan‐using behavior may be used to fulfill other desires, such as to improve air quality and air freshness, rather than improving thermal comfort.

### 4.2 Physiological responses induced by the PCS

It was hypothesized that human thermoregulation can be exercised without compromising thermal comfort by using the designed PCS. We found no significant differences in the torso skin temperature between the two scenarios (Figure 4D). Skin temperature is an essential driver for thermoregulation,^69^ especially in cold conditions.^70^ The primary thermosensory body sites used for thermoregulation are the hairy skin of the torso and proximal extremities,^69^ in particular the abdomen,^71^ whereas the non‐hairy skin sites (such as palms and soles) provide limited feedback signal to the thermoregulatory system.^69^, ^72^ The designed PCS mainly heated up the non‐hairy skin (palm and sole) and resulted in no significant changes in the major hair skin (torso). Therefore, it is reasonable to assume that the whole‐body thermoregulatory responses are similar between the two scenarios. The results confirm this, with the exception of the lowest temperature condition (17°C). Indeed, the PCS did not significantly change the energy expenditure, substrate oxidation, physical activity in most of the cases, and merely resulted in slight changes in the whole‐body cardiovascular responses (2.2 bpm increase in heat rate, 1.5 mmHg decrease in systolic blood pressure, and 0.9 mmHg decrease in diastolic blood pressure, see Figure 5). At 17°C, energy expenditure was relatively high in the PCS scenario, possibly because of the increase in physical activity (Figure 6A, E).

Interestingly, the hand skin blood flow significantly increased at 23°C in the PCS scenario while no significant differences were found at 17, 19, 21 and 25°C (Figure 5E). Hand skin blood flow is modulated by local temperatures and mean body temperature,^73^ where the proximal body part (core body tissue) dominates this modulation.^74^, ^75^ The effect of local heating on hand skin blood flow depends on the existing level of whole‐body vascular tone.^76^, ^77^ In this study, the designed PCS did not alter the thermal state of the torso in the range of 17–21°C (Figure 4D), therefore, the ‘cool’ torso still modulated the whole‐body vasomotor tone and dominated the hand skin blood flow. However, at 23°C, the torso was at a near‐neutral thermal state, where the local heating exerts much more influence on local skin blood flow. At 25°C, the decreased use of local heating and/or increased use of head cooling may have resulted in a non‐significant change in blood flow. The studies by Caldwell^76^ and Spealman^77^ corroborate our results. They show that the local heating only slightly affects hand skin blood flow when the body is hypothermic while it substantially elevates the hand skin blood flow when the body is normothermic or hyperthermic.

**FIGURE 11:**
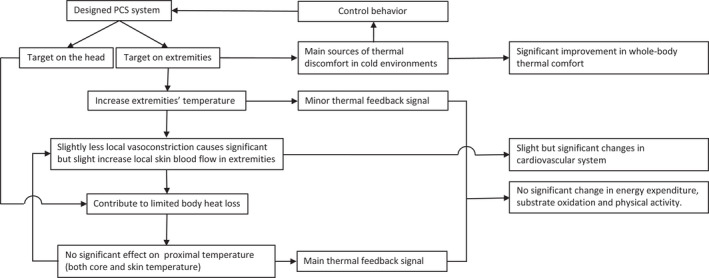
An explanatory framework for physiological responses by using the PCS

A possible framework is presented in Figure 11 to explain the physiological responses by using the PCS. The PCS manipulates the local temperature of hands and feet, decreases local vasoconstriction and slightly increases average by‐scenario skin blood flow in the extremities. It substantially mitigates the local thermal discomfort of extremities, resulting in considerable improvement in whole‐body thermal comfort. However, considering a relatively small heating area and a slight increase of extremities skin blood flow, the PCS only provides limited compensation to the whole‐body heat loss. Therefore, it does not affect the proximal thermal state, which dominates the whole‐body vasomotor tone and attenuates the effect of local heating on local skin blood flow. Conversely, the attenuated effect further constrains the heat transportation from extremities to the body (torso). On the other hand, at 25°C, the PCS slightly decreases head skin temperature and may also marginally contribute to the body heat loss. As a result, whole‐body thermoregulation does not deviate much between the two scenarios in terms of cardiovascular responses, metabolic response and physical activity responses, and hence, thermal stimulation by the dynamic ambient temperature may be maintained. An equivalence test based on the 90% confidence interval can test whether an effect is meaningful or not by assuming a meaningful threshold.^78^ If the 90% confidence interval does not contain the threshold, it can be concluded that for an alpha value of 0.05, an effect is statistically smaller than the meaningful threshold.^78^ As a meaningful threshold for an effect may differ among disciplines, Table S1 provides the 90% confidence interval of the differences in physiological responses between the two scenarios, to help readers to judge the meaning of those differences.

### 4.3 Health indications

Increasing human metabolism can offset the excessive energy intake combating the prevalence of obesity. Studies have shown that mild cold (16–19°C) can increase human energy expenditure.^79^, ^80^, ^81^ In contrast, our results show an even lower energy expenditure at 17°C in the NOPCS scenario compared to 23°C (Figure 6A). Differences in physical activity and circadian rhythm effects may explain this inconsistency. Firstly, the difference in physical activity may mask the real effect of mild cold on energy expenditure. On average, physical activity at 17°C in NOPCS scenario was lower than at 23°C (Figure 6E). Secondly, the circadian rhythm also plays a role. In our study, the 17°C occurred in the morning while 23°C occurred in the afternoon. Studies^82^, ^83^ report that energy expenditure is higher in the afternoon compared to the morning. This circadian‐rhythm‐induced increase at 23°C may override a cold‐induced increase at 17°C. Considering the evidence reported in the previous literature,^79^, ^80^, ^81^ we would expect higher energy expenditure in the mild cold (17–19°C) compared to neutral thermal conditions with other factors controlled. Our results indicate that, in most cases, the PCS did not affect the energy expenditure in comparison to the no‐PCS scenario (even higher at 17°C due to increased physical activity Figure 6). Therefore, using the designed PCS in mild cold conditions may retain cold‐induced thermogenesis, combating the prevalence of overweight. More importantly, realistic indoor temperature conditions (17–19°C) with long‐term exposures increase non‐shivering thermogenesis,^84^ even result in weight loss^84^ and affect insulin sensitivity.^85^ Our results suggest that the use of the PCS mitigated thermal discomfort and did not substantially reduce the human body's thermoregulatory effort in an ambient temperature range of 17–25°C. Therefore, the designed PCS combined with mildly cold conditions may possibly benefit metabolic health without compromising thermal comfort. Moreover, the results also suggest other health implications of the designed PCS. The first is the improved air quality perception and air freshness perception by using the PCS under slightly warm ambient thermal conditions, which is in accordance with previous studies.^66^, ^86^ Secondly, we also show that the ventilation element of the PCS could reduce eye strain at 25°C. Finally, the designed PCS boosted pleasure and arousal over the entire ambient thermal range of 17–25°C. Besides metabolic health, those results also demonstrate that the PCS may benefit psychological health.

### 4.4 Limitations

This study investigated the effects of a PCS that focuses on the extremities under ambient thermal conditions ranging between 17 and 25°C in a well‐controlled lab setting. However, there are two limitations. Firstly, this study was unable to demonstrate the effect of the PCS in warm conditions, because the highest imposed ambient temperature was 25°C (slightly warm). Further studies are needed to demonstrate if the thermoregulatory system can be stimulated under warmer ambient conditions without compromising thermal comfort. Secondly, the effect of the PCS on thermal perceptions at 17°C might be underestimated as the PCS just ran for about 10–20 min when the first questionnaire was filled at 17°C. The PCS may not have had sufficient time to warm up the extremities as no significant change in distal skin temperature was found at 17°C between the two scenarios (Figure 4A). With longer usage of the PCS, a greater improvement in thermal comfort at 17°C could be expected.

## 5 CONCLUSIONS

This paper has validated the hypothesis that human thermoregulation can be stimulated without compromising thermal comfort using a PCS design that targets only the extremities and the head. The main conclusions are as follows:

1. The designed PCS significantly improved thermal perceptions in a moderately drifting ambient temperature range of 17–23°C.
2. The designed PCS did not reduce whole‐body thermoregulatory responses in the drifting temperature range from 17–25°C. The torso skin temperature, underarm‐finger temperature gradients, energy expenditure, substrate oxidations and physical activity were generally similar between PCS and NOPCS scenarios in most cases.
3. The designed PCS slightly influenced cardiovascular responses (2.2 bpm increase in heat rate, 1.5 mmHg decrease in systolic blood pressure, 0.9 mmHg decrease in diastolic blood pressure and 8% increase in hand skin blood flow)
4. The designed PCS boosted pleasure and arousal. Although the PCS did not improve thermal comfort at 25°C, it significantly improved air quality and air freshness perceptions and mitigated eye strain at 25°C.

Overall, the results show that in a temperature drift human thermoregulation can be stimulated without compromising thermal comfort by using a PCS that only targets the extremities in cold conditions. The designed PCS may provide a great potential for the future to create a healthy, comfortable and energy‐efficient built environment.

## CONFLICT OF INTEREST

The authors have no conflict of interest to declare.

## AUTHOR CONTRIBUTIONS

W.L Conceptualization, Methodology, Investigation, Writing‐original draft, Writing‐review & editing; R.K Conceptualization, Methodology, Writing‐review & editing; Y.d.K Conceptualization, Methodology, Funding acquisition, Writing‐review & editing, Supervision; P.R Investigation, Writing‐review & editing; W.v.M.L Conceptualization, Methodology, Funding acquisition, Writing‐review & editing, Supervision.

### PEER REVIEW

The peer review history for this article is available at https://publons.com/publon/10.1111/ina.12951.

[Correction added on 20 December, after first online publication: Peer review history statement has been added.]

## Supporting information

Supplementary Material
