# Supplementary material for: The effects of a novel personal comfort system on thermal comfort, physiology and perceived indoor environmental quality, and its health implications ‐ Stimulating human thermoregulation without compromising thermal comfort
Source: Indoor Air. 2021 Nov 1;32(1):e12951. doi: 10.1111/ina.12951 (PMC9298036; doi:10.1111/ina.12951)
Supplement: Supplementary file 1 — Supplementary Material [file INA-32-0-s001.docx]

**Figure S1.** Visual analogue scales for measuring thermal perceptions a) thermal sensation scale b) thermal comfort scale c) thermal preference scale

**Figure S2.** Scales for self-perceived air quality, emotion and eye-related symptom. a) air quality scale b) air freshness scale c) SAM pleasure scale d) SAM arousal scale e) eye-related symptom scales.


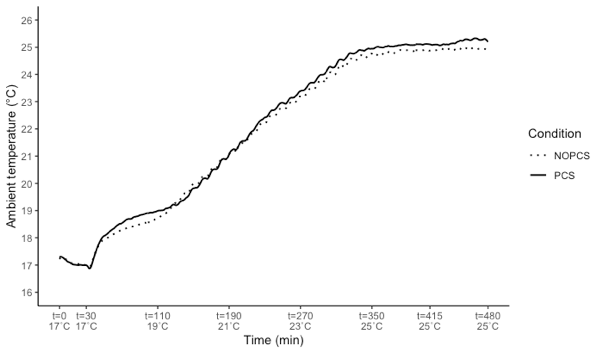


**Figure S3.** Average ambient air temperature


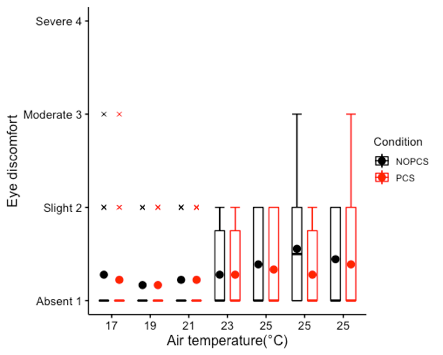

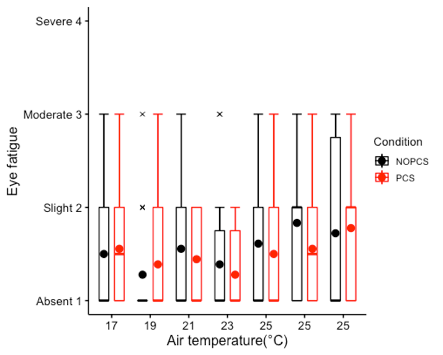


a) b)

**Figure S4.** Eye-related symptoms: a) Eye discomfort over time, d) eye fatigue over time.


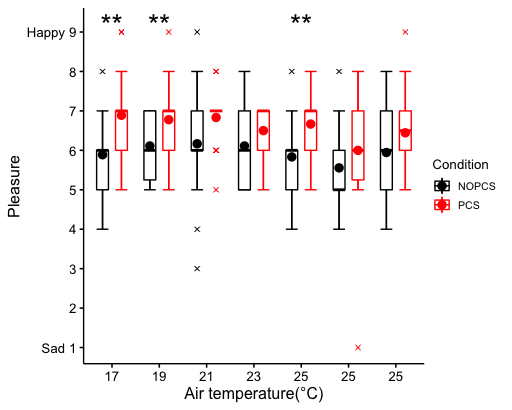

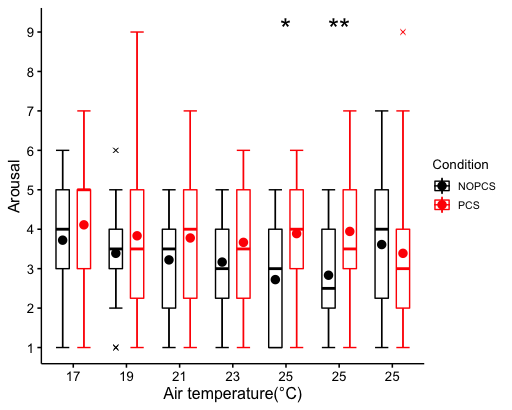


**Figure S5.** Affective states over time: a) pleasure, b) arousal.

**Table S1**. The differences in physiological responses between the two scenarios. (the PCS scenario compared to the NOPCS scenario)

| Parameters | Estimated mean | Standard error | 90% confidence interval |
| --- | --- | --- | --- |
| Torso skin temperature (˚C) | 0.0144 | 0.0483 | (-0.065, 0.094) |
| Underarm finger gradient (˚C) | 0.221 | 0.325 | (-0.312, 0.754) |
| Energy expenditure (KJ/min) | 0.021 | 0.062 | (-0.080, 0.123) |
| Lipid metabolism (g/min) | -0.0057 | 0.0042 | (-0.0126, 0.0012) |
| Carbohydrate metabolism (g/min) | 0.0157 | 0.009 | (0.00092, 0.03048) |
| Normalized hand skin blood flow | 0.081 | 0.038 | (0.018, 0.144) |
| Heart rate (bmp) | 2.2 | 0.5 | (1.36, 3.14) |
| Systolic blood pressure (mmHg) | -1.47 | 0.70 | (-2.62, -0.32) |
| Diastolic blood pressure (mmHg) | -0.95 | 0.49 | (-1.75, -0.15) |
| Intensity (count/min) † | 0.22 | / | (-1.10, 0.98) |

† note intensity data uses Wilcox test (non-parameter method). Therefore, the estimated mean for intensity is the pseudo median, no standard error is shown and the 90% confidence interval is based on median.
